# Reconstruction of a composite comparative map composed of ten legume genomes

**DOI:** 10.1007/s13258-016-0481-8

**Published:** 2016-10-21

**Authors:** Chaeyoung Lee, Dongwoon Yu, Hong-Kyu Choi, Ryan W. Kim

**Affiliations:** 1 Department of Medical Bioscience, Graduate School, Dong-A University, Nakdong-Daero 550-Beongil 37, Saha-Gu, Busan, 49315 Republic of Korea; 2 Department of Molecular Genetics, College of Natural Resources and Life Science, Dong-A University, Nakdong-Daero 550-Beongil 37, Saha-Gu, Busan, 49315 Republic of Korea; 3 SeqGenesis Inc., Gajeongbuk-Ro 96, Yuseong-Gu, Daejeon, 34111 Republic of Korea; 4 Korea Bioinformation Center, Korea Research Institute of Bioscience & Biotechnology, Gwahak-Ro 125 Yuseong-Gu, Daejeon, 34141 Republic of Korea

**Keywords:** Legumes, Comparative genomics, Gene-specific marker, Synteny

## Abstract

The Fabaceae (legume family) is the third largest and the second of agricultural importance among flowering plant groups. In this study, we report the reconstruction of a composite comparative map composed of ten legume genomes, including seven species from the galegoid clade (*Medicago truncatula*, *Medicago sativa*, *Lens culinaris, Pisum sativum*, *Lotus japonicus*, *Cicer arietinum*, *Vicia faba*) and three species from the phaseoloid clade (*Vigna radiata*, *Phaseolus vulgaris*, *Glycine max*). To accomplish this comparison, a total of 209 cross-species gene-derived markers were employed. The comparative analysis resulted in a single extensive genetic/genomic network composed of 93 chromosomes or linkage groups, from which 110 synteny blocks and other evolutionary events (e.g., 13 inversions) were identified. This comparative map also allowed us to deduce several large scale evolutionary events, such as chromosome fusion/fission, with which might explain differences in chromosome numbers among compared species or between the two clades. As a result, useful properties of cross-species genic markers were re-verified as an efficient tool for cross-species translation of genomic information, and similar approaches, combined with a high throughput bioinformatic marker design program, should be effective for applying the knowledge of trait-associated genes to other important crop species for breeding purposes. Here, we provide a basic comparative framework for the ten legume species, and expect to be usefully applied towards the crop improvement in legume breeding.

**Electronic supplementary material:**

The online version of this article (doi:10.1007/s13258-016-0481-8) contains supplementary material, which is available to authorized users.

## Introduction

The legume family (the Fabaceae or Leguminosae) is one of the most agro-economically important plant groups, second only to the grass family (the Poaceae or Gramineae), and contains 19,325 species and 727 genera, which is the third largest in the flowering plants (Lewis et al. 2005). Capability of fixing atmospheric nitrogen is an interesting and unique biological property of leguminous plants through symbiotic interaction with soil-borne Rhizobium bacteria. Traditionally, the Fabaceae is divided into three subfamilies, Caesalpionoideae, Mimosoideae and Papilionoideae. Of these, the Papilionodeae subfamily is the largest (approximately 14,000 species within 476 genera), known to have evolved relatively recently, which is monophyletic, and includes most of important cultivated legume crops. Almost all the cultivated grain legumes are derived from members of two clades within the Papilionoideae, galegoid (temperate or cool season legumes: barrel medic [*Medicago truncatula*, a legume model], alfalfa [*Medicago sativa*], pea [*Pisum sativum*], broad bean [*Vicia faba*], lentil [*Lens culinaris*]) and phaseoloid/milletioid (tropical legumes: soybean [*Glycine max*], common bean [*Phaseolus vulgaris*], mungbean [*Vigna radiata*], cowpea [*Vigna unguiculata*], adzuki bean [*Vigna angularis*], pigeon pea [*Cajanus cajan*]). In addition to grain legumes for human food, legume crops serve for a diverse array of utilities, such as forage for animal feed, oilseed, medicine and agroforestry (Singh et al. 2007). Legumes are economically important because numerous commercial products are manufactured using these crops including cosmetics, pharmaceuticals, soap, resins, paints and lubricants.

Historically, many different types of molecular markers have been developed and used for many applications, including marker-assisted breeding, phylogenetics/systematics, molecular ecology, forensics and diagnostics (Poczai et al. 2013), all of which depend on polymorphisms that can be analyzed by proper techniques. Traditionally, any random nucleotide variations found in mapping parents were employed for purposes of constructing genetic map and massive development of genetic markers. Such examples typically include restriction fragment length polymorphism (RFLP), amplified fragment length polymorphism (AFLP), random amplified polymorphic DNA (RAPD) and simple sequence repeat (SSR) markers. However, these techniques are commonly species-specific, which means that markers developed in one species can not readily cross-work in other species, mainly due to high sequence variations in randomly selected polymorphic regions. Such a demerit can be properly compensated by using gene-derived sequences whose genomic regions are relatively more conserved than in intergenic regions. This strategy was experimentally proved by designing PCR primer pairs within exon regions aligned with orthologous gene counterparts of compared species (Choi et al. 2004a, b). Advantage of such cross-species gene-derived markers can be applied for broader utilities in genome mapping and comparative analysis largely due to the translatability of marker information among different, but related, species.

**Fig. 1:**
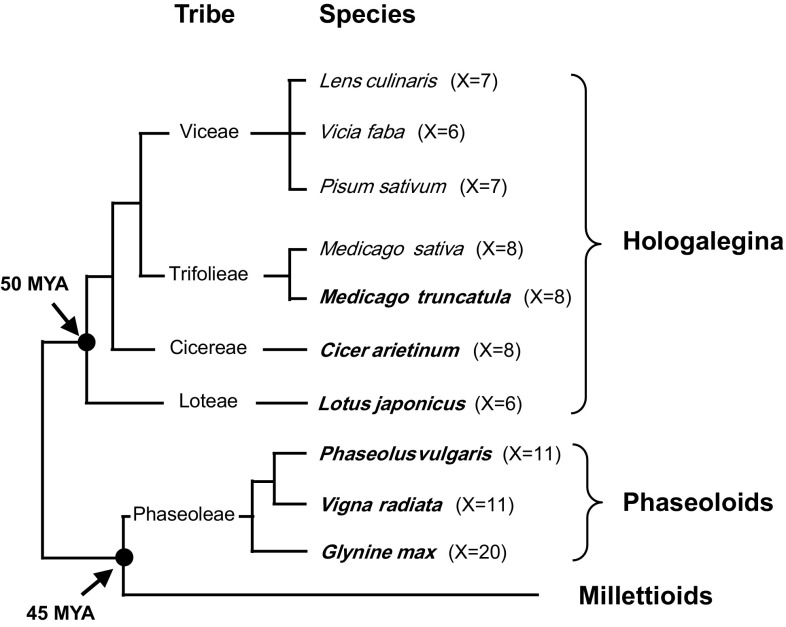
Taxonomic relationships of ten legume species used in this study. These species prevalently occur in five tribes within two major clades, hologalegina and phaseoloid clades. Of these, six species whose draft genome sequences have been reported are highlighted in *bold character*. *X* basic chromosome numbers; *MYA* million years ago

In this study, we aimed to integrate pre-existing genetic and genomic information from a total of ten legume genomes (for their phylogenetic relationship, see Fig. 1), and to construct a comparative genomic framework across a broad span of legume species. Although more detailed comparisons with the whole genome sequences, but with smaller number of legume species, were reported (Varshney et al. 2013), this study should be the first report for a composite comparative map containing the broadest set of legume genomes.

## Materials and methods

### Data resources for genetic/genomic mapping

To reconstruct the comparative genetic map composed of ten legume species, following data for genetic maps and marker information were employed: *M. truncatula* and *M. sativa*—Choi et al. 2004a; *L. japonicas*, *P. sativum*, *G. max*, *V. radiata*, *P. vulgaris* and *L. japonicus*—Choi et al. 2004b; *V. faba*—Ellwood et al. 2008; *L. culinaris*—Phan et al. 2006. Of these, genetic maps of four recently sequenced species were updated with the whole genome information for corresponding marker’s genomic positions and annotations by referring to the latest version of following genome databases: *M. truncatula*—JCVI (http://www.jcvi.org/cms/research/groups/plant-genomics/ ) v4.0; *G. max* and *P. vulgaris*—Phytozome DB (https://phytozome.jgi.doe.gov/) v9.0 and v1.0, respectively; *V. radiata*—SNU (Seoul National University) Plant Genomics DB (http://plantgenomics.snu.ac.kr/).

### Marker information and reconstruction of composite comparative genetic map

In the cases that genetic maps for each legume species were available and cross-species genic markers were used, relevant information was employed in a straightforward manner for the comparative genetic map. For four legume species having its whole genome sequences with reliable level of draft genome information (i.e., *M. truncatula*, *G. max, P. vulgaris* and *V. radiata*), maps were redrawn by locating genomic positions of cross-species markers. Genetic map of *M. truncatula*, a representative legume model with relatively simple genome structure, was used as the central genome for the comparative mapping throughout this study. To ensure precise genomic positions of individual markers and orthology of gene-based markers, the BlastN homology search was used for the *M. truncatula* and then a combination of homology searches (BlastN and tBlastX) was employed to define correct orthologous gene loci for other species. For cross-species translation of genic markers, the orthology of candidate genes was evaluated only when E-values of the homology search were <E^−50^. In addition, the accuracy of homology-based identification of cross-species orthologous genes was reconfirmed using in-house-programed electronic PCR (e-PCR). In order for the e-PCR, PCR primer pair sequences of *M. truncatula* were used and searched in other fully sequenced legume genomes (i.e., *G. max*, *P. vulgaris* and *V. radiata*). Wherever possible, positioning of orthologous genic markers on the genetic map were finally determined when results of both the homology search and the e-PCR were consistent with each other. After completing individual genetic maps for ten legume species, the maps were juxtaposed and integrated with each other, wherever possible, according to their relative closeness in phylogenetic distances. Collinear genic markers were represented by lines and synteny blocks were called based on collinearity of multiple markers within the syntenic regions.

## Results

### Basic genome information and phylogenetic relationships of compared legume species

In this study, we used a total of ten legume species, seven from the galegoid clade and three from the phaseoloid clade (Fig. 1). Their genomic information is summarized in Table 1. This comparative analysis included a broad range of genomes, ranging from 333 (the smallest genome of mung bean) to ~13,000 Mbp (the largest one of broad bean), which is approximately 39 times difference in the genome sizes. In basic chromosome numbers (X), it seems that ‘X = 6–8’ predominate in the galegoid legumes while ‘X = 11–20’ in the phaseoloid legumes (Table 1), implicating some level of chromosomal events, such as chromosome fusion and/or fission, during evolutionary divergence from common ancestor of these two clades. Among these legumes, soybean is particularly known as diploidized paleo-allo-tetraploid genome (Schmutz et al. 2010). Gene numbers are predicted relatively accurately for the whole genome-sequenced species ranging from 22,368 genes for the mung bean to 56,044 genes for the soybean, which is more than double in the gene context. Until recently, six legume genomes have been fully sequenced and their draft genome information has been reported (Table 1). In this study, genomic data for four species (*M. truncatula*, *G. max*, *P. vulgaris* and *V. radiata*) were employed, but other two (*L. japonicus* and *C. arietinum*) were not included because their genomes were relatively recently sequenced and genomic information was less reliable enough to accurately position genomic loci for each of the cross-species markers.

**Table 1:** Genomic information of ten legume species used in this study

Species name	Common name	Genome size (Mbp)	Chr. No.	Gene number	Remark	Reference
*Medicaog truncatula*	Barrel medic	470	2n = 2x = 16	50,894	WGS	Young et al. 2011
*Medicago sativa*	Alfafa	830–860	2n = 4x = 32	NA	–	Bauchan and Hossain 2001
*Pisum sativum*	Pea	4300	2n = 2x = 14	NA	–	Franssen et al. 2011
*Vicia faba*	Broad bean	~13,000	2n = 2x = 12	NA	–	Ellwood et al. 2008
*Lens culinaris*	Lentil	~4000	2n = 2x = 14	NA	–	Arumuganathan and Earle 1991
*Cicer arietinum*	Chickpea	864	2n = 2x = 16	28,269	WGS	Varshney et al. 2013
*Lotus japonicus*	Bird’s-foot trefoil	471	2n = 2x = 12	39,735	WGS	Sato et al. 2008
*Vigna radiata*	Mung bean	333	2n = 2x = 22	22,368	WGS	Kang et al. 2014
*Glycine max*	Soybean	1115	2n = 2x = 40	56,044	WGS	Schmutz et al. 2010
*Phaseolus vulgaris*	Common bean	625	2n = 2x = 22	38,482	WGS	Schmutz et al. 2014

*NA* Not available, *WGS* Whole genome sequencing completed

### Reconstruction of genetic/genomic maps and comparative analysis

**Fig. 2:**
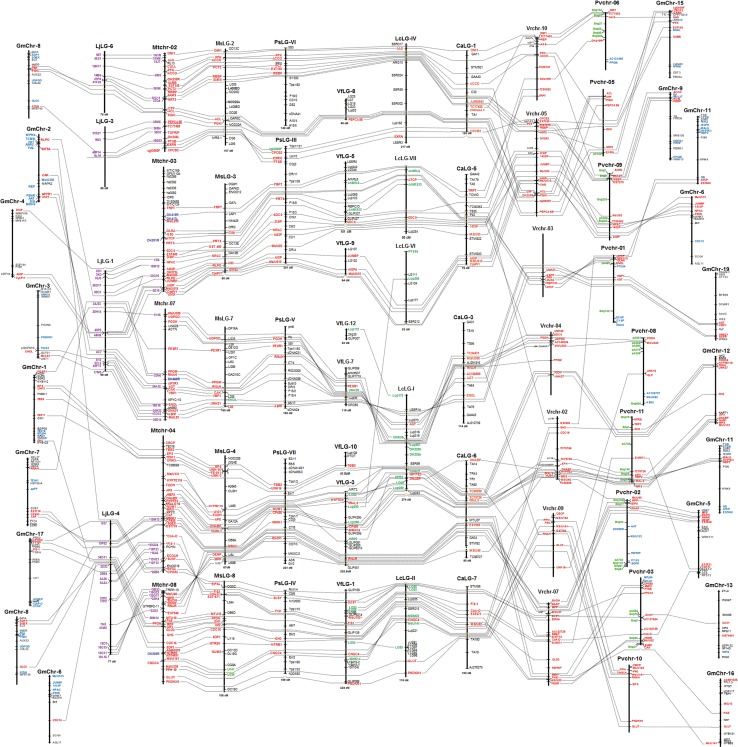
Macrosyntenic composite comparative map with reference to *M. truncatula* chromosomes 1, 5 and 6 (for remaining part of the comparative map, see Fig. S1). Cross-species translated markers are denoted by *bold lettering*. Predicted marker positions, but unmapped in genetic map, are extrapolated only when the collinearity is conserved in neighboring regions, and denoted by *dotted semicircle line*. Species names are as follows: *Mt*, *M. truncatula*; *Ms*, *M. sativa*; *Gm*, *G. max*, *Lj*, *L. japonicus*. *Ps*, *P. sativum*; *Vf*, *V. faba*; *Lc*, *L. culinaris*; *Ca*, *C. arietinum*; *Vr*, *V. radiata*; *Pv*, *P. vulgaris*. *Chr* chromosome, *LG* linkage group

**Fig. 3:**
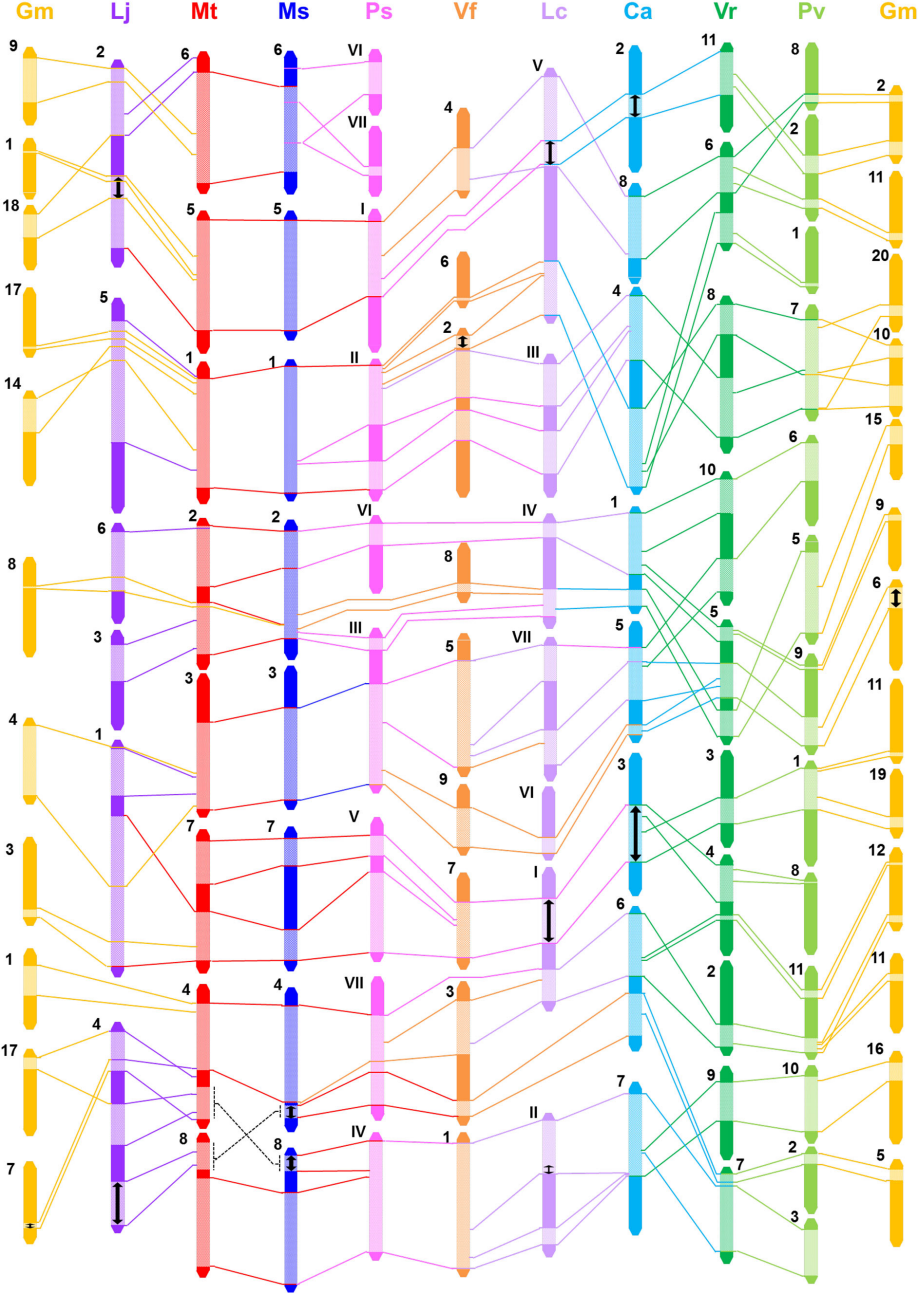
A simplified macrosyntenic relationships among ten legume species. Abbreviations for species names are the same as in Fig. 2. Sizes of chromosome/linkage group and synteny blocks are drawn to scale for each species, but not across species. *Lines* and *arrows* denote as follows: *solid lines* postulated rearrangement; *double headed arrow* postulated inversions

For purposes of conducting map-based comparative analysis, genetic maps for each of ten legume species were reconstituted using core gene-derived comparative markers (Table S1) and juxtaposed in parallel with each other. To facilitate revelation of syntenic relationships, individual maps were ordered, wherever possible, according to their phylogenetic relatedness. A total of 209 cross-species markers played a pivotal role in revealing syntenic relationships across these legume genomes. In all cases, *M. truncatula* genome played a central role for this comparative mapping, within which included a broad array of species composed of 6–20 chromosomes and 39 times variation in genome sizes. Despite these genomic diversities and limited number of markers, the cross-species genic markers could identify a total of 110 synteny blocks with various sizes across ten legume genomes in comparison and some chromosomal rearrangements as well. Details of composite genetic/genomic comparisons are demonstrated in Fig. 2 and Fig. S1. These composite comparative maps were further simplified to assist block-by-block identification of shared cross-genome syntenies. Intriguingly, a total of 93 chromosomes or linkage groups (refer to Table 1) from the entirety of ten legume genomes could be integrated into a single genetic map network (Fig. 3). Relevant marker information within the shared synteny blocks are shown in Table S2. Based on these data, it seems obvious, as naturally expected, that similarity in genomic structures of compared legume species increases in proportion to the phylogenetic closeness. In other words, we could find larger, on average, synteny blocks in between galegoid legumes, compared to ones with distantly related legumes in phaseoloid clade (Figs. 2, 3). For example, almost entire chromosome 1 of *M. truncatula* is syntenic with *M. sativa* LG-1, and divided into two large blocks in genomes of *P. sativum* LG-II, *V. faba* LG-2, *L. culinaris* LG-III and *C. arietinum* LG-IV (Fig. 3). In contrast, the same synteny blocks found in the galegoid legumes show more fragmental patterns in the phaseoloid legumes and represented by relatively smaller number of shared markers. The genome-wide cross-species syntenic relationships are summarized in Table 2, and the data should be useful to discover chromosome- and/or LG-level collinearities and to infer some genomic events by which might have occurred within the context of these compared species during the evolutionary pathways. For example, *M. truncatula* chromosome 1, as the nodal genome of this study, showed the relatively simplest chromosome level collinearities, almost one-to-one relationship with other legume genomes except for the *G. max*, which was predictable due to the paleo-tetraploidy nature of its genome structure. MtChr-6 is relatively poor in the number of mapped markers, and thus syntenies could not be extensively analyzed. This result is consistent with previous observation that MtChr-6 is relatively rich in heterochromatic DNA regions and lacks in transcribed genes (Choi et al. 2004a, b; Kulikova et al. 2001). Instead, it was found that MtChr-6 was enriched largely with resistance gene analogs (Young et al. 2011; Zhu et al. 2002). Among others, two legumes belonging to the same genus, *M. truncatula* and *M. sativa*, showed the most extensive synteny to each other, which would be easily predictable. However, one exceptional chromosomal rearrangement, terminal reciprocal/inverted translocation, was identified between MtChr-4/MtChr-8 and MsLG-4/MsLG-8 (Fig. 3, Fig. S1). In addition, 13 inversion events were identified among all these legume genomes, which could be a structural modulator in legume genome evolution (Fig. 3).

**Table 2:** Conserved chromosome/LG information of syntenic regions

Species^a^	Conserved chromosome/linkage blocks^b^							
Mt	1	2	3	4	5	6	7	8
Ms	1^L^	2^L^	3^L^	4^L^, 8^S^	5^L^	6^L^	7^L^	4^S^, 8^L^
Ps	II^L^	III^S^, VI^M^	III^L^	IV^M^, VII^L^	I^L^	VI^M^, VII^S^	V^L^	IV^L^, VII^S^
Vf	6^S^, 2^L:^	NA	5^L^, 9^L^	1^S^, 3^S^	4^L^	NA	7^L^	1^L^, 3^S^
Lc	III^M^	IV^M^	VI^M^, VII^L^	I^M^	V^M^	NA	I^M^	II^M^
Ca	4^S^	1^L^	5^L^	6^M^	2^S^, 8^L^	NA	3^M^	6^M^, 7^L^
Lj	5^L^	3^M^, 6^L^	1^M^	4^M^	2^M^	2^M^	1^M^	4^S^
Vr	4^M^	5^M^, 10^M^	5^M^, 10^M^	2^M^, 4^S^, 7^S^	6^M^, 11^L^	NA	3^M^, 4^M^	7^L^, 9^L^
Pv	7^M^	5^L^, 6^L^, 9^S^	9^M^	11^M^	2^M^	NA	1^M^, 8^S^	2^S^, 3^L^, 10^L^
Gm	14^S^, 17^S^, 20^S^	8^S^, 15^M^	4^L^, 9^S^	1^M^, 11^S^, 12^S^	1^S^, 2^S^, 11^S^	9^L^	3^S^,11^S^,19^S^	13^S^, 16^M^

^a^Species names are the same as in Fig. 2

^b^Conserved block sizes: *L* large; *M* moderate; *S* small

## Discussion

Utility of comparative analysis is based on the idea that evolutionarily related species are diverged from their common ancestor and conserved genome synteny can be effectively translated from a well-studied species to other less characterized genomes. Such an idea has been articulated in many plant families, including the Brassicaceae (Schranz et al. 2006, 2007), Poaceae (Gale and Devos 1998; Mayer et al. 2011) as well as the Fabaceae (Choi et al. 2004b; Hougaard et al. 2008), and even across multiple families (Abrouk et al. 2010; Tang et al. 2008b). Such cross-species translation of genomic information can be effectively accomplished using orthologous genes or genomic loci that have shared evolutionary pathways. However, comparative analysis of genomes among different species is not simple to precisely define orthologous genes or genomic loci in a straightforward manner, and rather often complicated by gene duplication, recurring polyploidy and extensive genome rearrangement (Tang et al. 2008a). Recent whole genome sequencing and analyses have revealed a general history of genome duplications followed by gene and/or genomic level erosion, which also may mislead researchers to biased results of comparative analyses (Kaul et al. 2000; Schmutz et al. 2010). Due to such genomic complexity, reliably determining orthology of shared genes between compared genomes should be the key to robustness for the genome comparative analysis.

In the case of legume family, six species, in total, have been reported for their fully sequenced draft genomes until now (Table 1), which might be an enough number of species to offer the basis for genome research in this family. However, there are still numerous crop legumes of agricultural importance and with a long history of breeding that remain orphan with limited molecular and genomic characterization. For relatively less studied crop genomes, projection of genomic and/or gene information obtained from well-studied species is essential to infer function of individual genes and evolutionary relationships within the context of genomic structures. Moreover, such translated information can be practiced in crop breeding for the trait improvement of agricultural interests. Naturally, the translational accuracy of genome synteny is higher among closely related species, and this notion was re-proved in this study. It was also evidenced that differences in genome sizes did not significantly disrupt the macro-syntenic relationships (Choi et al. 2004b), as shown in the cases of species with large genomes such as pea, broad bean and lentil, all of which are members of the tribe Viceae (Fig. 1). This result indicates that particularly the Viceae tribe seems to have experienced genome expansion and related genomic events, typically mediated by mobile genomic elements, predominantly occurred in intergenic regions, which occupy the vast majority of genomes in most cases of higher eukaryotic organisms.

The genome comparative analyses were represented either by actual chromosomes for fully sequenced genomes or by linkage groups of genetic maps, all of which correspond to each other, except for only one species. The genetic map of broad bean (*V. faba*) is not yet populated densely with a sufficient number of genetic markers, thereby still consisting of 12 fragmental linkage groups (Ellwood et al. 2008) compared to actual six chromosomes (Table 1). Set aside of this species, simplified view of comparative genome structures among other 9 legume species (Fig. 3; Table 2) should offer an opportunity to infer possible evolutionary events how these genomes have shaped into current genome structures. Within the context of genome information used in this study, they are different from each other in chromosome number, size and ploidy. Legumes belonging to the galegoid clade (X = 6–8) are predominant with relatively smaller numbers of chromosomes, while ones from the phaseoloid clade (X = 11–20) have more chromosome numbers (Fig. 1; Table 1). Based on phylogenetic relationship and estimated divergence time (Fig. 1), it is assumed that a common ancestral genome with smaller basic chromosome number evolved towards a direction of increasing the chromosome numbers. This implicate that certain large scale chromosomal changes, such as chromosome fusion or fission, had occurred during divergence of these legume species in between the two clades. For example, macrosyntenies found in MtChr-5/6 versus LjLG-2 and MtChr-4/8 versus LjLG-4 (Figs. 2, S1; Table 2) may propose the evolutionary mechanism of fusion/fission, followed by inversions of genomic blocks in part, among these chromosomes in comparison and explain a major cause of the chromosome number reduction in *L. japonicus* genome. Similarly, cross-clade macrosyntenic correlations typically found between *M. truncatula* and *V. radiata*, for examples MtChr-8 versus VrChr-7/9 (Fig. S1) and MtChr-5 versus VrChr-6/11 (Fig. 2), provides a wealth of genomic evidences for the chromosomal fission contributing to the increase of chromosome numbers in the phaseoloid legume genomes. In addition to such large scale chromosomal events, a diverse array of genomic changes and reshufflings were revealed in this comparative analysis, all of which might have played a combined role in establishing the current status of compared ten legume genomes. However, this study was conducted using a limited number of genetic markers, only 209 cross-species genic markers, and thus may offer only a rough idea on plausible evolutionary pathways within these genomes. As the NGS technology has rapidly advanced in recent years, the whole genome sequencing (WGS) has become relatively much easier and faster, and subsequently the comparative analyses of genomes currently tend to be more dependent on fully sequenced genome information. Actually, WGS-based comparative analyses were performed, at least in part, with fully sequenced draft genome information for four legume species including *M. truncatula*, *L. japonicus*, pigeonpea (*Cajanus cajan*) and chickpea (Varshney et al. 2013). This study revealed a lot broader genomic conservations represented by 110 synteny blocks that were identified using 15,441 orthologous groups, which is currently the most comprehensive WGS-based comparative genome analysis within the Fabaceae. It is expected that more extensive genome level comparisons will become available as the WGS information for more legume species will be produced in the future, thereafter providing a deeper insight into the genomic correlation and evolutionary history among important legume genomes.

Although production of the WGS information by the NGS techniques has now become the experimental routine for many researchers and laboratories, it is practically true that establishment of a well-defined reference genome and general application of the NGS methods to a diverse array of crop species are still limited. Such situation can be further aggravated particularly in orphan crops with very large genomes, for instance the Viceae legumes used in this study. In such cases, genetic map-based comparative analysis will be able to play effective roles in translating genome information between related species. Reconstruction of the composite comparative map in this study could be made by using shared genic markers, which were developed before and thereafter used to map other legume species by multiple researchers, and by integrating genetic maps for the ten legume species. This approach could be achieved due to the attribute of cross-genome translatability of gene-derived markers, by which can reliably find orthologous gene loci across many different, but related, species. In order to design the cross-species genic markers, one needs a fair amount of genomic information at least from two related species and must carefully design to satisfy the required conditions for cross-species PCR amplification. In recent years, a bioinformatic platform, called ‘CSGM (cross-species genic marker) Designer (http://tgil.dau.ac.kr/ CSGMdesigner)’, was developed with an aim to facilitate high throughput design of the cross-species markers (Kim et al. 2015). This design program has following advantageous features; (i) linked directly with the legume reference genome database, (ii) enables rapid search and retrieval of target gene information for the marker design, (iii) visualizes PCR primer candidates by graphics, (iv) can pre-verify cross-species amplifiability based on the electronic PCR. If combined with genomic information relating to trait-associated genes gained from the resequencing and GWAS data of well-studied species, such bioinformatic marker design platform will be able to accelerate the development of functionally associated gene-derived markers and allow us to more reliably translate the inter-species genomic information into less-studied orphan, but agriculturally important, species for molecular crop improvement.

In summary, beneficial features of the gene-based markers for the cross-species translation of orthologous genomic information were re-evaluated and re-verified through reconstructing an extended composite comparative map composed of ten important model or crop legume species. The resulting outcome is a single, but extensive, comparative network of genetic maps, which consists of 93 chromosomes/linkage groups from the ten legume genomes. This genetic map network would presumably be one of the broadest, but not the most comprehensive, comparative analyses that have been reported until now. It is anticipated that the results and relevant information should offer a useful framework to gain insights into the structural correlations and evolution-related knowledge in legume genomes, and may provide practical information that can be used for the legume crop improvement.

## Electronic supplementary material

Below is the link to the electronic supplementary material.

Supplementary material 1 (DOCX 322 kb)

Supplementary material 2 (XLSX 63 kb)

Supplementary material 3 (XLSX 73 kb)
