# Supplementary figures and images for: Reconstruction of a composite comparative map composed of ten legume genomes
Source: Genes Genomics. 2016 Oct 21;39(1):111–9. doi: 10.1007/s13258-016-0481-8 (PMC5196017; doi:10.1007/s13258-016-0481-8)

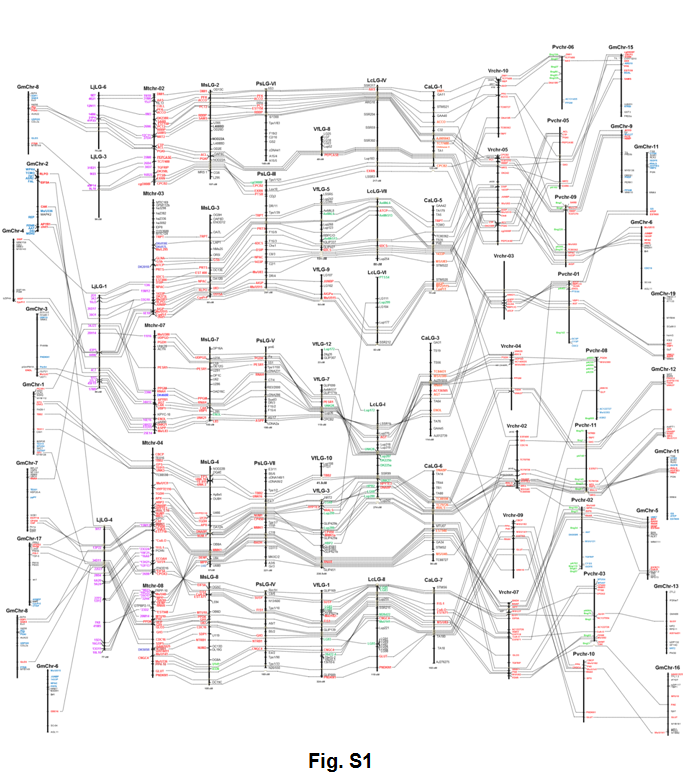

Supplement: Supplementary file 1 — Supplementary material 1 (DOCX 322 kb) [file 13258_2016_481_MOESM1_ESM.docx]
